# Satisfaction in conventional acrylic complete denture patient with and without denture liners - a systematic review

**DOI:** 10.11604/pamj.2022.42.296.33035

**Published:** 2022-08-22

**Authors:** Jayesh Shinde, Tushar Mowade, Priya Gupta, Rahul Tekale, Neelam Pande, Kalyani Deshmukh, Twinkle Lokhande, Usha Radke

**Affiliations:** 1 Department of Prosthodontics, VSPM Dental College and Research College, Nagpur, India

**Keywords:** Denture liners, patient satisfaction, systematic review

## Abstract

To make a new complete denture, a series of steps is needed, and it is a time-consuming process. Which is not possible in every patient due to financial and medical reasons. The old denture can be relined by denture liners in less clinical and laboratory steps and can successfully increase the efficiency of ill-fitting dentures however, patient satisfaction with denture liners is doubtful. This systematic review was organized from the preferred reporting items for systematic reviews' checklist, and the methods were registered on the international prospective register of systematic reviews (PROSPERO- CRD42020210227). The main systematic review is to assess satisfaction in convectional acrylic complete denture with or without denture liners. Search engines such as PubMed, Science Direct, Cochrane, Ovid, and Google Scholar were used to extract information. The risk of bias was measured with the help of the Cochrane collaboration tool. Initially we found 1711 articles out of which 6 were finalised as per PICO criteria. Patient satisfaction was evaluated by using a visual analogue scale and questionnaire method, which shows denture liners group has more patient satisfaction. Acrylic denture liner and silicon denture liner shows better patient satisfaction compared to the conventional acrylic denture. Among denture liners, there is no significant difference.

## Introduction

The worldwide elderly population is growing rapidly, and although many among them have natural teeth preserved, significant numbers undergo from loss of teeth [1,2]. The complication that appears from edentulism is difficulty in biting food which results in poor health, anaesthetic appearance, and difficulty in speech, all of which lead to physical impairment [3,4] which affects the overall health of patient [5-7]. In many edentulous patients, a conventional complete denture is one of the main choices [8] while, implants are more efficient still implants cannot worthwhile key for many patients for the reason of medical problems, psychological problems, bone quality, quantity and economic status has a vital role in the treatment plan [9-11]. Complete denture considers satisfactory by edentulous patients. Even a complete denture after some time shows some problems like sufficient stability, insufficient retention and pain during mastication [12]. This problem is mostly seen in a patient with an atrophic mandibular ridge and thin mucosa [13]. Continuous Residual ridge resorption causes inadequate support of complete denture. Loose complete denture causes difficulty in eating, speaking and induce anxiety in patients which force them to withdraw from social activity and reduces their quality of life [14,15]. New denture fabrication can increase the quality of life and patient satisfaction by increasing the proper fit of the denture and proper border seal, which increases the retention and support [16-18].

New denture fabrication leads to further periodic visits to the dentists, this can be costly. But, those economic problems have restricted the approach to the dental care [19]. Soft liners have been recommended as an affordable solution for such cases [20]. Liners are non-invasive and more economical compared to new dentures [21-23]. Soft liners work as a shock absorber because of their resilience nature which distributes functional stress, making prostheses comfortable to wear [9-11]. Acceptance of prostheses by patients can be determined by their degree of satisfaction [24-25]. Thus, this systematic review aims to determine the satisfaction of patients with the conventional acrylic complete denture and compare it with the denture liner group. In addition, assess the results of studies investigating patient satisfaction among denture lining materials and conventional acrylic dentures.

## Methods

**Reporting format:** protocol of the study is registered in Prospero with ID - CRD42020210227. Moher *et al*. [25] and Cochrane collaboration [26] were used for reporting format. This Systematic review was conducted to address the specific PICO Criteria [27,28].

**Search strategy and inclusion:** literature published in the years from January 1990 to December 2020 was sought. Articles in the English language were searched. PubMed, Google Scholar, science direct, Cochrane were used to search the articles. Manual searching in grey literature was also done. The search for grey literature was carried out in the Open Grey database. All full-text articles are included.

**Selection of studies:** authors (J.S., P.G.) independently extracted data. Pre-determined inclusion criteria were used to find the articles. Studies under author, country, study participants, study design, gender, age, clinical parameters and main outcome. A second author checked the information collected.

**Inclusion criteria:** articles were included in this systematic review if they fulfil the following criteria. All excluded studies with the reason given in (Table 1) [29-42].

**Table 1:** excluded studies

Author	Reason for exclusion
Valentini F *et al*. [29]	Surface roughness
Krunic N *et al*. [30]	Oral heath quality of life
Kimoto S *et al*. [31]	Longevity of denture
Kimoto S *et al*. [32]	Clinical effect
Kimoto S *et al*. [33]	Masticatory ability
Kimoto S *et al*. [34]	Chewing ability
Villar A *et al*. [35]	Clinical evaluation
Mutluay MM *et al*. [36]	Clinical performance
Furokawa S *et al*. [37]	Pain sensation
Ohkawa S *et al*. [38]	Clinical evaluation
Uysal H *et al*. [39]	Cushion adhesive
Means CR *et al*. [40]	Clinical evaluation
Koronis S *et al*. [41]	Cushion adhesive
Goncalves TM *et al*. [42]	Denture adhesive

**Study type:** full text randomised control studies which are published from January 1990 to December 2020 in peer-review journals, primary journals, be on a human subject.

**Participants:** edentulous patient wearing an acrylic complete denture.

**Outcome measures:** patient satisfaction was measured with a visual analogue scale and questionnaire method.

**Risk of bias:** revised Cochrane risk of bias tool for randomized trials dated October 9, 2018 were used for risk of bias assessment for randomised control trial [43]. the risk of bias assessment of all randomised control trials is shown in Table 2 [44-49].

**Data extracted:** the following data were extracted from included randomised control trials: author, year, jaw, control, intervention, follow-up, patient satisfaction by visual analogue scale, and patient satisfaction by questionnaire. These data were compiled in standard a table.

**Characteristics of included study:** the characteristic of all included study is given in (Table 3) [44-49]. All the studies were randomised control trials. Patient satisfaction by visual analogue (VAS) scale and questionnaire method was measured.

## Current status of knowledge

**Search strategy for identification of studies:** PICO criteria were followed. The focused question was “In edentulous patients does the use of conventional acrylic denture with and without denture liner result in a difference in patient satisfaction”.

PICO format: population: edentulous patients wearing conventional acrylic complete dentures with and without denture liner. Intervention: denture liners. Comparison: conventional acrylic denture and acrylic denture with a denture liner. Outcome: patient satisfaction by visual analogue scale and questionnaire.

PICO criteria were followed for search strategy. The search terms used were “Complete denture” OR “Edentulous patients” AND “Denture liners” OR “Soft liners” AND “Denture without liner” OR “Conventional denture” OR “Acrylic denture” AND “Patient satisfaction” OR “Chewing efficiency” OR “Speaking efficiency” OR “Cleaning” OR “Stability of denture” OR “Retention of denture” OR “Denture esthetics” OR “General satisfaction” OR “Resilient denture liner”. A combination of all these terms was used for the desired research. A manual hand search was also performed to find more related studies. Articles published from January 1991 to December 2020 were searched. Reviewers (J.S., P.G.) reviewed all the articles independently. After going through all the articles, inclusion and exclusion criteria followed.

**Figure 1:**
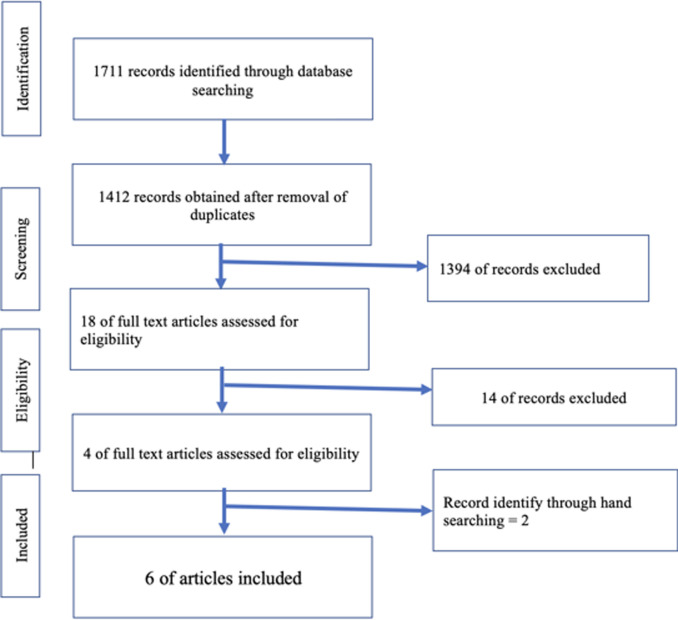
flowchart

Initially, 1711 articles were found with duplicates removed and 1412 screened. After title and abstract check, 18 were screened. Two independent authors (J.S. and P.G.) assessed the eligibility of articles, of which 14 were excluded (Table 1). Four articles were found eligible for the study, and two articles were added by hand searching. A total of six articles were analysed in the systematic review (Figure 1). Meta-analysis was not possible as high heterogenicity was present in studies.

**Inclusion criteria:** in vivo study, human trials, randomized clinical trials, studies published in English, healthy adults, no limits on the number of patients were placed, denture liner with the acrylic denture in any form and a control group as a conventional acrylic denture.

**Exclusion criteria:** in vitro study, non-human, non-English, studies on a patient with systematic diseases, case reports and case series, retrospective studies, reviews.

**Figure 2:**
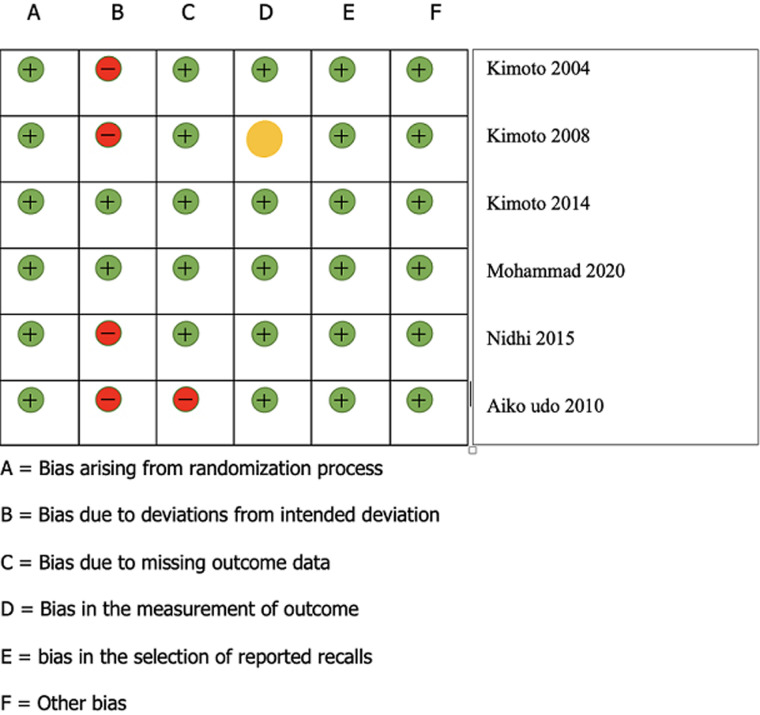
risk of bias summary for included studies according to Cochrane risk of bias domain for randomised control trials

**Quality assessment and risk of bias:** revised Cochrane risk of bias tool [27] was used for risk of bias evaluation. Two reviewers (J.S., P.G.) perform the quality assessment of the study. Cochrane collaboration guideline was used to make six domains and probability of bias assessment. Domains are: 1) Year 2) Randomization 3) Deviation from intervention 4) Missing outcome data 5) Measurements 6) Reported recalls. During the judgement low and high risks of bias were specified, while “Unclear” meant an uncertain risk of bias. A study was grouped as “low risk of bias” when all the aspects were of low risk of bias and as high or uncertain when one or more aspects were of “High or Unclear risk of bias” (Table 2, Figure 2).

**Table 2:** risk of bias assessment for included studies

Study	Year	Randomization	Deviation from intervention	Missing outcome data	Measurement	Reported recall	Other bias
Kimoto *et al*. [44]	2008	Low	High	Low	Unclear	Low	Low
Kimoto *et al*. [45]	2014	Low	low	Low	Low	Low	Low
Mohammad *et al*. [46]	2020	Low	Low	low	Low	Low	Low
Nidhi *et al*. [47]	2015	Low	High	Low	Low	Low	Low
Kimoto *et al*. [48]	2004	Low	High	Low	Low	Low	Low
Aiko udo *et al*. [49]	2010	Low	high	high	low	low	Low

**Characteristic of study:** the characteristics of all included studies are listed in (Table 3). All studies were randomised control trials. Patient satisfaction is checked with a visual analogue scale and questionnaire method.

**Table 3:** characteristics of studies

Sr. No	Author	Year	Jaw	Control	Intervention	Follow up	Patient satisfaction by Visual analogue scale	Patient satisfaction by questionnaire
1	Kimoto *et al*. [44]	2008	Mandible	Acrylic denture	Acrylic denture liner	2 years	Satisfaction rating higher for acrylic denture liner. Significant difference is seen in maxillary denture for satisfaction with speaking.	
2	Kimoto *et al*. [45]	2014	Maxilla and Mandible	Acrylic denture	Acrylic denture liner	2 years	Significantly higher in acrylic denture liner. Chewing satisfaction for maxillary denture is significantly higher.	
3	Mohamad *et al*. [46]	2019	Mandible	Acrylic dentures	Acrylic denture liner and Silicon denture liner	3 months	Silicon denture liner and acrylic denture liner higher satisfaction than acrylic denture. No difference between silicon and acrylic denture liner.	
4	Nidhi mangtani *et al*. [47]	2015	Mandible	Acrylic denture	Acrylic denture liner	1 year	-	Significant difference seen in satisfaction with chewing and comfort in denture liner group
5	Kimoto *et al*. [48]	2004	Mandible	Acrylic denture	Silicone denture liner	3 months	Not significant	
6	Aiko udo Yamakawa *et al*. [49]	2009	Maxillary complete denture	Office denture liner	Home denture liner	4 days	Not significant	

**Patient satisfaction with a denture liner result:** data formed as author, year, jaw, control, intervention, patient satisfaction by visual analogue scale, and patient satisfaction by questionnaire (Table 3). Two studies from Kimoto and one study from Mohamad shows that patient were more satisfied with the denture liner group [44-46]. Even Nidhi magnate compared the acrylic resilient soft denture liner (RLL) and acrylic denture with a questionnaire and found that chewing efficiency and comfort were in the denture liner group [47]. One of the studies from Kimoto shows no difference between patient satisfaction in denture liner and conventional acrylic group [48]. Aiko Udo-Yamakawa and Yasuhiko Kawai compare home denture liners and office denture liners and found that office and home denture liners show 10 - 20 percent greater than before in VAS [49]. Still, this value was lower compared to new-made acrylic complete denture [50].

## Discussion

In the dental material and bacteriological field, resilient denture liners have been studied for decades [51-54]. Despite the clinician's effort to make a good denture and the patient's desire for a satisfactory complete denture, a patient having resorbed ridge and thin gingiva biotype face pain during chewing. These truths inspire researchers to start research in denture liners [55]. Kimoto compares permanent acrylic resilient liner dentures (RLD) with conventional heat-activated acrylic resin dentures (ARD) with 100 mm VAS and found denture liners have more satisfaction compared to acrylic dentures. Addon denture liner in mandible comparing other outcomes found that denture functions such as chewing, speaking, stability, retention, and aesthetics significantly correlated with ratings of comfort and pain. This shows the perception of pain and patient satisfaction were greatly changed from denture delivery to the first appointment recall [44].

Kimoto also compares acrylic resilient denture liner (ARLD) with the conventional acrylic denture (CARD) with 100 mm VAS, and they found that general satisfaction, satisfaction with chewing, and satisfaction with speaking is significantly higher in ARLD group [45]. Nidhi magnate and colleagues compare the acrylic resilient soft denture liner (RLL) with acrylic denture with a questionnaire and found satisfaction with chewing and satisfaction of comfort were more with denture liner group [47]. In all the above studies, the acrylic denture liner had better patient satisfaction compared to the conventional acrylic denture [44,45]. In vitro studies showed that the stress distribution effect of resilient denture liner can be the reason behind less pain. As there is less pain in the denture liner group, the comfort will be more compared to the conventional acrylic complete denture group [9,10]. Furthermore, if a complete denture patient experienced pain hence they will waver to talk which will result in decreased satisfaction with speaking [45]. It was also reported that sensory feedback of masticatory function might be controlled by mechanoreceptors which are present in the denture supporting area, so if the patient has pain then the masticatory satisfaction will also be less [56]. Considering that mastication affects oral health-related quality of life [57-58]. It is believable that chewing rating and speaking disturb general satisfaction hence we can say that general satisfaction is more with acrylic denture liner group surrounding oral structure controls the mandibular denture so when in pain patient cannot tolerate the denture causes decrease stability and retention [45,59,60]. Many studies show that patients with resilient denture liners have better retention and stability [61]. Pain also has a negative relation with aesthetic too [44].

Mohamad and colleagues compared silicone resilient denture liners (SRL), acrylic resilient denture liners (ARL) and conventional acrylic denture (CAR) with 100 mm VAS and concluded that patients were more satisfied with acrylic denture liners and silicon denture liners compared to the conventional acrylic denture. Hygiene maintenance was high in the CAR group. Patient satisfaction was insignificant in SRL and ARL groups. Better load distribution and the cushioning effect of soft liners in comparison to the hard nature of conventional acrylic dentures could be the reason behind higher patient satisfaction with denture liners [46]. Moreover, the increased retention and stability reported for dentures with resilient liners further improved chewing and speaking ability [44]. Low surface hardness and consequently reduced resistance to scratches, porosity, and high water sorption in addition to the detrimental effects of common denture liners are the reasons behind less satisfaction with hygiene maintenance [62,63]. Kimoto and colleagues compared the silicone denture liner with the conventional acrylic denture by 100 mm VAS and found no differences between patients' subjective ratings between them. This result was different from the previous study done by Murata *et al*. and the reason might be samples are from a large spectrum. This difference would reasonably explain the disparity in patient ratings between the two studies, and it is observed that there will be equivalence between resilient and acrylic resin materials by measuring with VAS when a wide spectrum of the subject was considered [10,48].

Aiko Udo-Yamakawa and Yasuhiko Kawai compare home (HR) and office (OR) denture liners with 100 mm VAS and found no significant difference in patient satisfaction between them. Both the materials show a 10 - 20 mm increase in VAS, but this value was still lower than VAS rating of the newly fabricated denture. The long-term effect of these materials was not assessed because long-term follow up would be unethical as per product instruction to extend beyond 4 days [49]. Studies show that there was no difference found between the acrylic denture liner and silicon denture liner by Mohamed and Aiko Udo-Yamakawa didn´t find any difference between home and office denture liners [46,49] and one of the answers to this can be VAS is not as much of sensitive in making dissimilarities between really comparable appliances [64]. Denture liner group produce considerable improvement in patient satisfaction compared with conventional hard based dentures. However, viscoelastic property of denture liners also plays an important role in increasing patient satisfaction. It is found that viscoelastic property is better in acrylic denture liners compare to silicone denture liners. Furthermore, this property also helps to absorb energy, equalise force, and directly prevent the transmission of force to tissues, absorb energy, and prevent transmission of force to the denture bearing tissues, ultimately cushioning effect will reduce the pain [11].

## Conclusion

We have concluded that, acrylic denture liner and silicon denture liner show better patient satisfaction compared to the conventional acrylic denture. There is no difference in patient satisfaction between acrylic denture liner and silicon denture liner. Home and office denture liners show no difference in patient satisfaction.

### What is known about this topic

- Denture liners are non-invasive and more economical compare to the fabrication of new complete dentures;
- Soft liners work as a shock absorber because of their resilience nature which distributes functional stress making prostheses comfortable to wear.

### What this study adds

- In this systematic review, patient satisfaction with denture liner is evaluated, and we found that the denture liner group has more satisfaction.
